# Cloning and Functional Characterization of a Novel Brevinin-1-Type Peptide from *Sylvirana guentheri* with Anticancer Activity

**DOI:** 10.3390/cimb47080673

**Published:** 2025-08-20

**Authors:** Huyen Thi La, Quynh Bach Thi Nhu, Hai Manh Tran, Huyen Thi Ngo, Phuc Minh Thi Le, Hanh Hong Hoang, Linh Trong Nguyen, Dat Tien Nguyen, Thanh Quang Ta

**Affiliations:** 1 Institute of Biology, Vietnam Academy of Science and Technology, Hanoi 100000, Vietnam; 2 Vietnam Academy of Science and Technology, Graduate University of Science and Technology, Hanoi 100000, Vietnam; 3 Faculty of Medical Technology, Hai Phong University of Medicine and Pharmacy, Hai Phong 180000, Vietnam; btnquynh@hpmu.edu.vn; 4 Center for High Technology Research and Development, Vietnam Academy of Science and Technology, Hanoi 100000, Vietnam; 5 Bac Thang Long Hospital, Hanoi 100000, Vietnam

**Keywords:** antimicrobial peptides, brevinin-1 E8.13, *Sylvirana guentheri*, anticancer activity, skin secretion, CAFLUX HepG2 cells

## Abstract

Despite significant medical advancements, two major health challenges persist: antibiotic resistance in microbial pathogens and drug resistance in cancer cells. To address these issues, research has increasingly focused on discovering novel natural compounds with dual antimicrobial and anticancer activities. Among such candidates, antimicrobial peptides (AMPs) have attracted attention due to their ability to selectively target microbial and cancer cells while exhibiting minimal toxicity toward normal cells. Although Vietnam possesses rich biodiversity, including a wide range of Anura species, studies on AMPs from these organisms remain limited. In this study, a novel AMP, brevinin-1 E8.13, was identified from the skin secretion of *Sylvirana guentheri*, a frog species native to Vietnam. The brevinin-1 E8.13 peptide was successfully cloned, sequenced, and chemically synthesized. Functional assays revealed that brevinin-1 E8.13 possesses strong antibacterial activity against Staphylococcus aureus and exerts significant antiproliferative effects on various human cancer cell lines, including A549 (lung), AGS (gastric), Jurkat (leukemia), HCT116 (colorectal), HL60 (leukemia), and HepG2 (liver). The peptide demonstrated moderate to potent cytotoxic activity, with IC_50_ values ranging from 7.5 to 14.8 μM, depending on the cell type. Notably, brevinin-1 E8.13 exhibited low cytotoxicity toward normal human dermal fibroblast (HDF) cells and even promoted cell proliferation at lower concentrations. Furthermore, Chemically Activated Fluorescent Expression (CAFLUX) bioassay results confirmed that the peptide significantly downregulated *Cyp1a1* gene expression in HepG2 cells. Collectively, these findings highlight the therapeutic potential of brevinin-1 E8.13 as a dual-function antimicrobial and anticancer agent derived from the skin secretion of *Sylvirana guentheri*.

## 1. Introduction

In recent years, an increase in cancer-related mortality has been observed, despite significant advancements in cancer treatment. This phenomenon is primarily attributed to the ability of cancer cells to develop resistance to existing anticancer drugs. Consequently, the development of anticancer agents with novel mechanisms of action has garnered considerable research interest. Among such agents, antimicrobial peptides have attracted attention due to their broad-spectrum cytotoxic activity against cancer cells and minimal toxicity to normal mammalian cells [1].

AMPs are small, biologically active molecules characterized by a net positive charge and amphipathic properties, possessing both hydrophobic and hydrophilic regions. These peptides, which vary in amino acid composition and length (ranging from six to 100 residues), play a critical role in innate immune defense mechanisms against microbial infections through diverse modes of action [2]. In contrast to conventional antibiotics, peptide antibiotics selectively target microbial cell membranes, which differ structurally from those of plant and animal cells. This selective targeting enables the disruption of microbial membranes without damaging host cells [3].

The brevinin-1 peptide family is recognized as one of the most extensively distributed AMP families, particularly prevalent among amphibians. These peptides exhibit broad-spectrum antimicrobial activity against various bacterial, fungal, and some viral pathogens. Their primary mechanism of action involves the disruption of microbial membrane integrity, resulting in cell lysis. Due to their widespread occurrence and potent antimicrobial properties, brevinin-1 peptides remain a focal point in the search for novel therapeutics targeting drug-resistant microbes [4]. Since the initial identification of brevinin-1 from Rana brevipoda porsa, a total of 985 brevinin-1 peptides have been documented and archived in relevant databases [5].

According to the Antimicrobial Peptide Database (APD), a total of 2619 AMPs have been cataloged to date. This includes 261 bacteriocins derived from bacteria, 4 AMPs from archaea, 7 from protists, 13 from fungi, 321 from plants, and 1972 host defense peptides from animals. Among these, 2169 have been classified as antibacterial, 172 as antiviral, 105 as anti-HIV, 959 as antifungal, 80 as antiparasitic, and 185 as anticancer peptides [6]. The APD continues to expand as updated findings contribute to this comprehensive repository.

Aiming to discover new peptides and identify novel biological activities, in this study, genes encoding peptides were cloned from the skin secretion of *Sylvirana guentheri*. Based on the identification of conserved motifs and structural characteristics, a novel bioactive peptide, Brevinin-1 E8.13, was identified. This peptide exhibited strong cytotoxic effects against cancer cell lines while showing minimal toxicity toward normal human cells. Moreover, a significant downregulation of Cyp1a1 expression was observed, as confirmed by the CAFLUX bioassay.

## 2. Materials and Methods

### 2.1. Strains, Cell Lines, and Reagents

Several human cancer cell lines, including A549, AGS, Jurkat, HCT116, HL60, and HepG2, were employed for the assessment of anticancer activity. Dulbecco’s Modified Eagle Medium (DMEM) and Roswell Park Memorial Institute (RPMI) medium, obtained from Gibco (Thermo Fisher Scientific, Waltham, MA, USA), were used for the culture of mammalian cells. The HepG2 CAFLUX cell line was developed by the Animal Cell Biotechnology Laboratory, Institute of Biotechnology, Vietnam Academy of Science and Technology. This cell line had been transfected with a genetic construct containing a dioxin-responsive elements (DREs)-containing promoter and an H2B-EGFP reporter gene (a human histone H2B gene with enhanced green fluorescent protein gene).

*T4 DNA ligase* and the *pCR2.1* cloning vector were purchased from Thermo Fisher Scientific (USA). Custom cloning primers were supplied by PhuSa Biochem (Can Tho, Vietnam). *Escherichia coli* DH5α (Thermo Fisher Scientific, USA) served as the host strain for both cloning and recombinant expression, and all bacterial cultures were grown in Luria–Bertani (LB) medium.

#### Isolation and Characterization of Antimicrobial Peptides from *Sylvirana guentheri* (Ha Giang, Vietnam)

Three adult *Sylvirana guentheri* frogs were used. Each animal was fully anesthetized with 0.1% MS-222 (tricaine methane-sulfonate) before gentle electrical stimulation (5 mA, 100 Hz, 140 ms pulse width) was applied to collect skin secretion [7]. After secretion harvesting, all frogs were revived and released at the capture site; no animals were sacrificed or harmed.

The collected secretion was immediately frozen in liquid nitrogen and pulverized. Total RNA was extracted with TRIzol^®^ Reagent (Invitrogen, Carlsbad, CA, USA), and complementary DNA (cDNA) was synthesized using oligo(dT) primers. Target cDNA fragments were amplified by PCR with the primer PepF (5′-ATGTTCTTRAAGAAAWCC-3′) under the following cycling conditions: 95 °C for 3 min; 30 cycles of 95 °C for 30 s, 42 °C for 30 s, and 72 °C for 30 s; final extension at 72 °C for 8 min.

Amplified products were ligated into the *pCR2.1* vector, transformed into *E. coli* DH5α, and plated on selective medium. Positive clones were verified by colony PCR and sequenced with an ABI 3100 Avant Genetic Analyzer (Applied Biosystems, Waltham, MA, USA).

The sequence was analyzed by Bioinformatic tools. NCBI-BLAST (https://blast.ncbi.nlm.nih.gov/Blast.cgi, accessed on 20 August 2024) was used to identify the region of the mature peptide by comparing this sequence with all the sequences recorded in GenBank. The 3D structure was built based on the model of Ressource Parisienne en BioInformatique Structurale (RPBS) and viewed by Jalview 2.11.4.0 and the Agadir algorithm was used to measure the percentage of Helical Content in different conditions.

### 2.2. Peptide Synthesis via Solid-Phase Peptide Synthesis (SPPS)

Based on the identified nucleotide sequence, the brevinin-1 E8.13 peptide and a control peptide, GA-K4AL (amino acid sequence: FAKWAFKWLKK-NH_2_, as previously reported [8]), were synthesized using standard Fmoc-based solid-phase peptide synthesis (SPPS). The synthesis followed established protocols as described in previous studies [7]. Briefly, peptides were assembled on Rink amide resin (loading capacity: 0.6 mmol/g) using an automated peptide synthesizer. Each Fmoc-protected amino acid was added sequentially at a fourfold molar excess and activated with O-Benzotriazole-N,N,N′,N′-tetramethyluronium hexafluorophosphate/1-hydroxybenzotriazole (HBTU/HOBt) and N,N-Diisopropylethylamine (DIPEA) in Dimethylformamide (DMF). Fmoc deprotection was performed using 20% piperidine in DMF.

Following chain assembly, peptides were cleaved from the resin using a cocktail of trifluoroacetic acid (TFA), triisopropylsilane (TIS), and water (95:2.5:2.5, *v*/*v*/*v*) for 2–3 h at room temperature. Crude peptides were precipitated with cold diethyl ether, lyophilized, and purified by reverse-phase high-performance liquid chromatography (RP-HPLC). Peptide purity and molecular identity were confirmed by analytical HPLC and matrix-assisted laser desorption/ionization time-of-flight mass spectrometry (MALDI-TOF MS), with a final purity ≥ 90%.

### 2.3. FTIR Spectral Analysis

Fourier-transform infrared (FTIR) spectroscopy was performed on lyophilized peptide samples using a Bruker Alpha II FTIR spectrometer (Ettlingen, Germany) equipped with an Attenuated Total Reflectance (ATR) probe. Approximately 2 mg of peptide powder was placed directly on the ATR crystal surface and gently pressed to ensure optimal contact. Spectra were recorded over the range of 4000–400 cm^−1^ with a resolution of 4 cm^−1^ and averaged over 32 scans. A background spectrum was collected prior to each measurement and automatically subtracted to minimize noise. Characteristic absorption peaks corresponding to functional group vibrations within the peptide structure were identified based on their wavenumber positions in the recorded spectra.

For the solution-state measurement, the peptide sample was dissolved in deionized water at a concentration of 1 mg/mL. A small volume of the peptide solution was directly applied onto the surface of the ATR (attenuated total reflectance) crystal of the FTIR instrument, and the measurement was conducted under the same conditions as for the solid-state sample.

### 2.4. Circular Dichroism (CD) Spectral Analysis

The CD spectrum of the peptide brevinin-1 E8.13 was measured to evaluate its secondary structure. The peptide was dissolved in deionized water at a concentration of 0.3 mg/mL. Measurements were performed using a ChiraScan™ Circular Dichroism Spectrometer (Applied Photophysics, Leatherhead, Surrey, UK) with a quartz cuvette of 1 mm path length. The CD spectra were recorded over a wavelength range of 180–260 nm using the default instrument settings recommended by the manufacturer (scanning speed, integration time, and bandwidth). Each sample was measured three consecutive times, and the solvent-corrected average spectrum was used for analysis. The resulting spectra were processed using the CDNN 2.1 (Circular Dichroism Neural Network) software to estimate the proportions of secondary structural elements, including α-helix, β-sheet, β-turn, and random coil.

### 2.5. Antibacterial Activity of Brevinin-1 E8.13 Peptide

The antibacterial activity of brevinin-1 E8.13 was evaluated against six bacterial strains, including two Gram-positive species (*Staphylococcus aureus* and *Bacillus subtilis*) and four Gram-negative species (*Escherichia coli*, *Acinetobacter baumannii*, *Klebsiella pneumoniae*, and *Pseudomonas aeruginosa*). All strains were isolated and identified by the Department of Microbiology, Thanh Nhan Hospital (Hanoi, Vietnam), and are currently maintained at the Laboratory of Cell Biotechnology and Bioassay, Institute of Biology, Vietnam Academy of Science and Technology (VAST).

Each bacterial strain was first cultured overnight in Luria–Bertani (LB) broth. Then, 100 μL of the overnight culture was transferred to 5 mL of fresh LB broth and incubated at 37 °C with shaking until reaching the logarithmic growth phase. Cultures were adjusted to a concentration of 10^6^ colony-forming units (CFU)/mL and dispensed into 96-well plates. The peptide was added at final concentrations ranging from 24.8 μM to 0.4 μM. After 24 h of incubation at 37 °C, bacterial growth was quantified by measuring optical density (OD) at 600 nm using a Synergy HT microplate reader (BioTek, Winooski, VT, USA). All concentrations were tested in triplicate.

### 2.6. Cytotoxic Effects of Brevinin-1 E8.13 Peptide on Cancer and Normal Cells

The cytotoxic effects of brevinin-1 E8.13 were evaluated in six human cancer cell lines: A549 (lung), AGS (stomach), Jurkat (blood), HCT116 (colon), HL60 (blood), and HepG2 (liver). All cell lines were cultured in either DMEM or RPMI-1640 medium supplemented with 10% fetal bovine serum (FBS) and 1% penicillin–streptomycin, under conditions of 5% CO_2_ at 37 °C.

The 3-(4,5-dimethylthiazol-2-yl)-2,5-diphenyltetrazolium bromide (MTT) assay was used to assess cell viability. Cells were seeded at a density of 10^4^ cells per well in 96-well plates and incubated at 37 °C. After 72 h of treatment with the peptide, 10 µL of MTT reagent (Merck, Darmstadt, Germany) in PBS, pH 7.0 was added to each well and incubated for 4 h. Following supernatant removal, 100 µL of dimethyl sulfoxide (DMSO, Merck, Germany) was added to dissolve the resulting formazan crystals. Absorbance was measured at 570 nm using a microplate reader. Each experimental condition was performed in triplicate. The cytotoxicity of the peptide was also evaluated in normal human dermal fibroblast cells using the same assay.

### 2.7. Hemolytic Activity Assay of Brevinin-1 E8.13

To assess the hemolytic activity of Brevinin-1 E8.13, 2 mL of peripheral blood was collected from a healthy adult volunteer with informed consent. Red blood cells (RBCs) were isolated by centrifugation at 1000× *g* for 10 min and washed three times with phosphate-buffered saline (PBS, pH 7.4). The final RBC suspension was adjusted to a concentration of 1 × 10^6^ cells per well.

The peptide was diluted in PBS to final concentrations of 2.5, 5, 20, 30, 40, and 50 µM. In a 96-well plate, 100 µL of RBC suspension was mixed with 100 µL of peptide solution per well. For controls, 0.1% Triton X-100 in PBS was used as a positive control (100% hemolysis), while PBS alone served as the negative control (0% hemolysis).

After incubation at room temperature (22–25 °C) for 1 h, the plate was centrifuged at 1000× *g* for 5 min. Then, 100 µL of the supernatant from each well was transferred to a new 96-well plate, and hemoglobin release was quantified by measuring absorbance from 410 to 600 nm using a Synergy HT multi-mode microplate reader (BioTek Instruments, Winooski, VT, USA). Hemolysis (%) was calculated using the following formula: Hemolysis (%) = [(A_sample − A_PBS)/(A_Triton X-100 − A_PBS)] × 100. All assays were conducted in triplicate.

### 2.8. Evaluation of the Effect of Brevinin-1 E8.13 on Cyp1a1 Expression via the CAFLUX Assay

The CAFLUX HepG2 cell line was seeded into 96-well plates at a density of 10^4^ cells per well in 100 µL of DMEM supplemented with 10% FBS and 1% penicillin–streptomycin. The assay was conducted in triplicate. After incubation for 24 h at 37 °C in a 5% CO_2_ atmosphere, the cells were treated with brevinin-1 E8.13 at concentrations of 5, 10, and 15 µM, followed by an additional 24 h incubation.

The expression of GFP was quantified by measuring fluorescence intensity using a Synergy HT microplate reader with excitation at 485 nm and emission at 515 nm. Fluorescence imaging was performed using a Nikon C1si confocal microscope [9].

### 2.9. Statistical Analysis

All experimental data were analyzed using SPSS 22 software (IBM, Armonk, NY, USA). Statistical significance between groups was determined using one-way analysis of variance (ANOVA), followed by Tukey’s Honestly Significant Difference (HSD) post-hoc test to determine pairwise differences between groups. A *p*-value of less than 0.05 was considered statistically significant. Data are presented as the mean of three independent experiments, with error bars representing the standard error of the mean (SEM).

### 2.10. Ethical Considerations

This study involved the collection of peripheral blood from a healthy adult volunteer solely for the purpose of evaluating hemolytic activity. Written informed consent was obtained prior to sample collection. The study protocol was reviewed and approved by the Institutional Review Board of the Vietnam Academy of Science and Technology (Approval Code: 4792/QD-BCT). All procedures involving human samples were carried out in accordance with applicable ethical guidelines and regulations.

## 3. Results

### 3.1. Isolation and Characterization of Peptides from the Skin Secretion of Sylvirana guentheri

**Figure 1:**
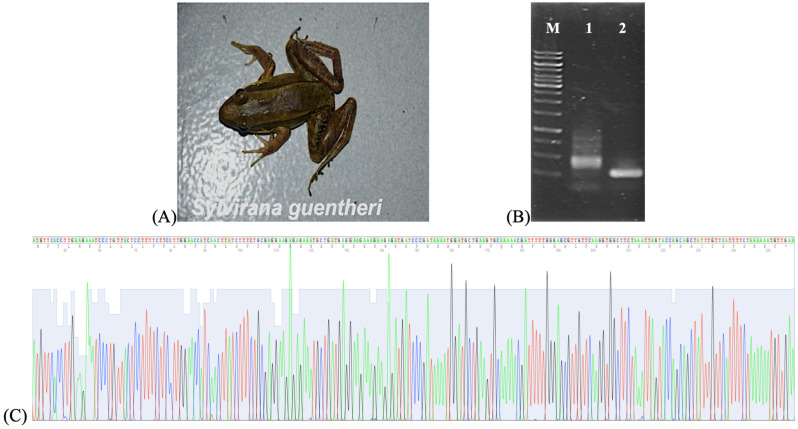
Results of isolation of peptides from the skin secretion of *Sylvirana guentheri*. (**A**) *Sylvirana guenther* used for peptide isolation *i*. (**B**) Agarose gel electrophoresis of PCR products with (**A**) PepF/Oligo T primers, M: Marker 1 Kb; 1: PCR products were inserted into the PCR2.1 vector; 2: PCR product with PepF/oligoT primers. (**C**) The gene cloning efficiency.

Total RNA was extracted from the skin secretion of *Sylvirana guentheri*, and its concentration and purity were assessed using a NanoDrop spectrophotometer (Thermo Fisher Scientific, USA). Complementary DNA (cDNA) was subsequently synthesized and used as the template for polymerase chain reaction (PCR) amplification with PepF/oligoT primers. The resulting PCR products were inserted into the pCR2.1 vector and transformed into *Escherichia coli* DH5α competent cells. The efficiency of gene cloning was evaluated by colony PCR using M13F/R primers, and selected recombinant vectors were subjected to DNA sequencing (Figure 1).

**Figure 2:**
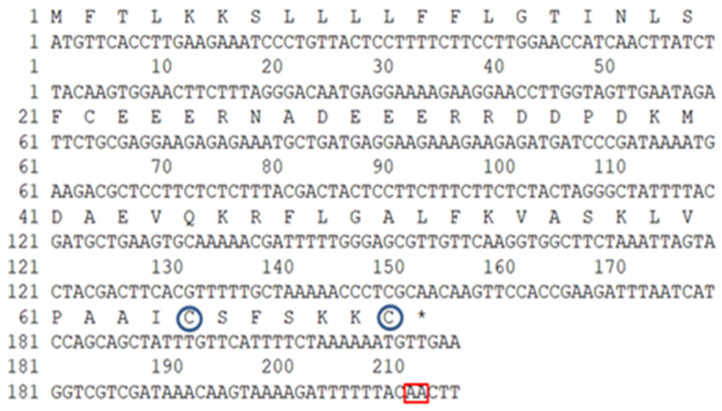
Sequences of DNA and polypeptide chain of brevini-1 E8.13 peptide. Red box: AA motif, blue circle: cysteine in motif Cys-5aa-Cys, and asterisk (*): stop codon.

Successful integration of the target DNA into the vector was confirmed by the observation of an increased PCR product size, relative to that of the original vector. This size increase indicated the presence of inserted DNA, thereby confirming the incorporation of peptide-encoding genes. One selected clone, designated E8.13, was sequenced, and the resulting nucleotide sequence was submitted to the NCBI GenBank under accession number UVJ64031 (Figure 2).

The open reading frame (ORF) was found to encode a total of 71 amino acid residues. The N-terminal region consisted of a 24-residue putative signal peptide, followed by a spacer domain rich in acidic amino acid residues, which terminated with a typical propeptide convertase processing site (-Lys-Arg- or -KR-). The mature antimicrobial peptide sequence was located at the C-terminal region. Additionally, two conserved motifs were identified: an amidated C-terminus and a Cys–5aa–Cys motif.

To facilitate further characterization, indices predictive of biological activity were calculated and analyzed using the Antimicrobial Peptide Database (Table 1).

The stability of the brevinin-1 E8.13 secondary structure under various conditions—such as different pH levels and temperatures—was assessed using the Agadir algorithm (Table 2). Under physiological conditions (37 °C, pH 7.0), the peptide was predicted to maintain a Helical Content of approximately 34–36%.

### 3.2. Synthesis and Structural Characterization of Brevinin-1 E8.13 and GA-K4AL

Two peptides—Brevinin-1 E8.13 and the control peptide GA-K4AL—were successfully synthesized using standard Fmoc-based solid-phase peptide synthesis. Following cleavage and purification, both peptides were evaluated for purity and molecular identity using reverse-phase high-performance liquid chromatography and matrix-assisted laser desorption/ionization time-of-flight mass spectrometry.

**Figure 3:**
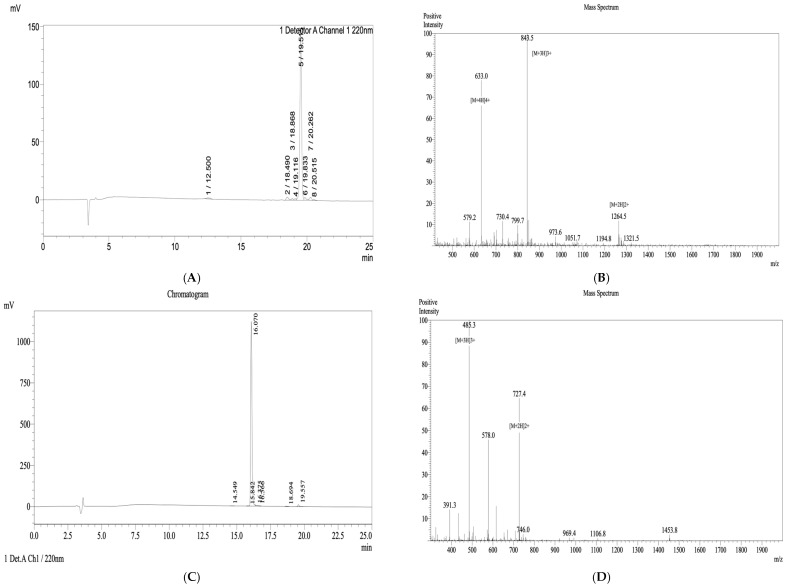
Analytical RP-HPLC chromatograms and MALDI-TOF mass spectra of synthetic peptides. (**A**,**C**) RP-HPLC chromatograms of Brevinin-1 E8.13 and GA-K4AL, respectively. (**B**,**D**) MALDI-TOF mass spectra of Brevinin-1 E8.13 and GA-K4AL, respectively.

The RP-HPLC chromatogram of Brevinin-1 E8.13 (Figure 3A) displayed a dominant peak at 19.52 min, accounting for 90.09% of the total area, indicating high purity. Similarly, the GA-K4AL peptide (Figure 3C) showed a sharp peak at 16.07 min, with a calculated purity of 97.899%. MALDI-TOF MS confirmed the molecular identity of both peptides. For Brevinin-1 E8.13, the observed molecular ion peak at *m*/*z* = 2527.5 was consistent with its theoretical mass of 2528.1 Da (Figure 3B). For GA-K4AL, the detected *m*/*z* value of 1452.9 closely matched its theoretical mass of 1452.79 Da (Figure 3D).

### 3.3. FTIR, CD Spectral Analysis

**Figure 4:**
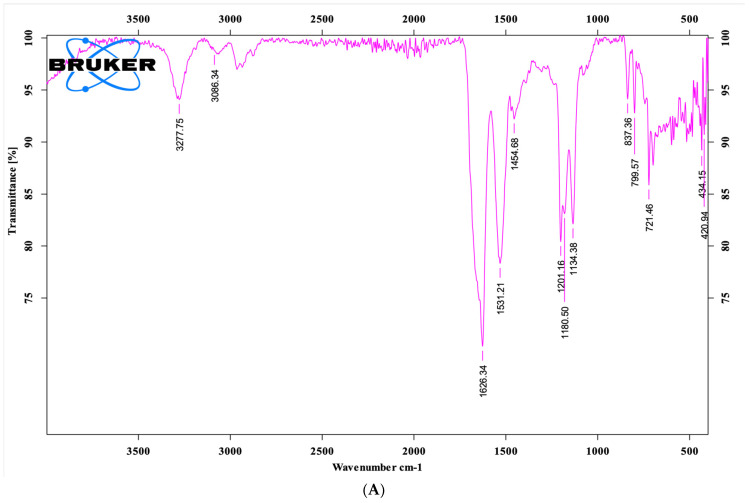

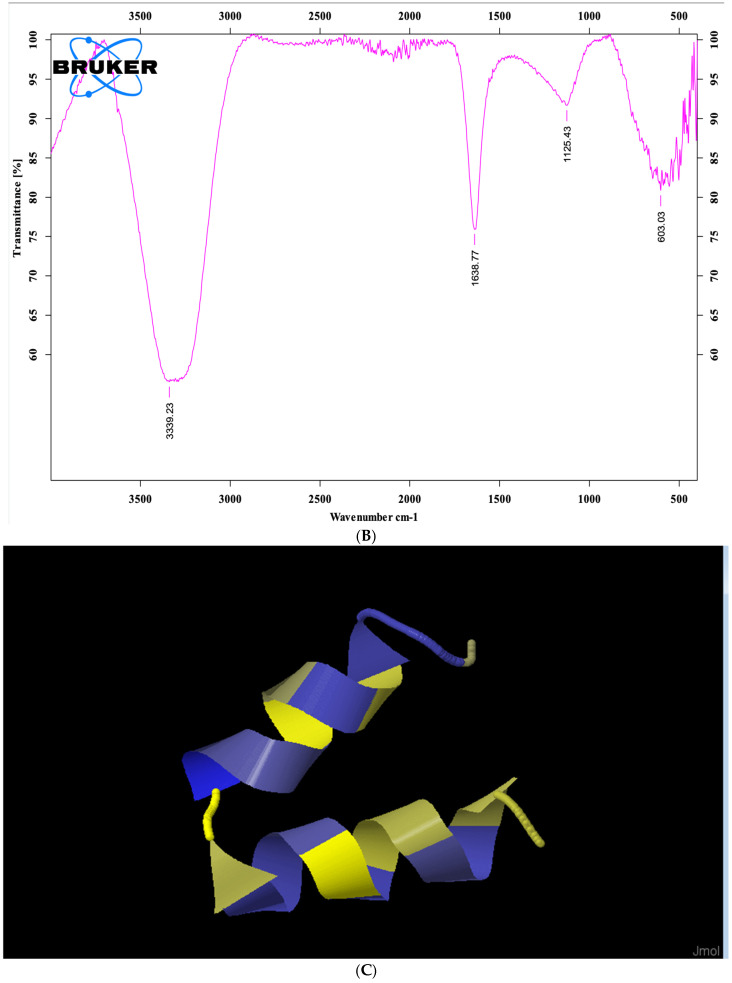

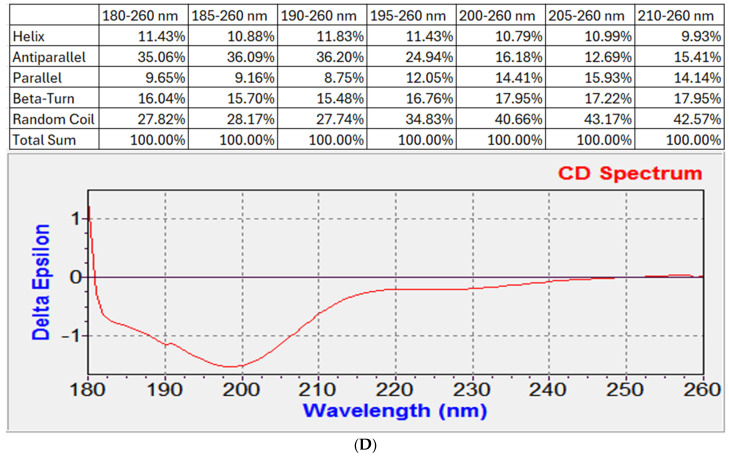
FTIR, CD spectrum and 3D-structure model of the brevinin-1 E8.13 peptide: (**A**) solid-state sample; (**B**) solution sample and (**C**) 3D-structure model; (**D**) CD spectrum.

The FTIR spectrum of the brevinin-1 E8.13 peptide (Figure 4) revealed characteristic absorption bands corresponding to the vibrations of the peptide backbone. In the solid state (Figure 4A), a prominent Amide I band appeared around 1626 cm^−1^, indicative of a dominant β-sheet structure. Additionally, a sharp N–H stretching band was observed at approximately 3277 cm^−1^, reflecting the formation of intermolecular hydrogen bonds. In contrast, the FTIR spectrum of the peptide in solution (Figure 4B) showed a shift in the Amide I band to approximately 1638.77 cm^−1^, suggesting the presence of a random coil structure, with a possibly low proportion of α-helix. The three-dimensional structure model of the peptide (Figure 4C), generated using RPBS predictions and visualized with Jmol, also indicated a mixed conformation of short α-helices and random coils, consistent with the FTIR findings. The CD spectrum (Figure 4D) of brevinin-1 E8.13 displayed a clear negative peak around 198–200 nm, with an upward trend beyond 210 nm, reflecting a characteristic mixed secondary structure. Quantitative analysis using CDNN 2.1 software revealed that the peptide’s predominant conformations were random coil (27.74%) and β-sheet (including antiparallel 36.2% and β-turn 15.48%), while the α-helix content was relatively low at 11.83% (based on the 190–260 nm range). This pattern was consistent across various analytical windows, confirming that the peptide adopts a flexible, non-uniform folded conformation, typical of short antimicrobial peptides rich in polar amino acids. These complementary results demonstrate that brevinin-1 E8.13 possesses a stable yet dynamic secondary structure—a key feature underlying its biological activity and membrane interaction potential.

### 3.4. Antibacterial Activity Assay

The antibacterial activity of brevinin-1 E8.13 is summarized in Table 3. Brevinin-1 E8.13 exhibited strong inhibitory effects against *Staphylococcus aureus*, with a minimum inhibitory concentration (MIC) of 1.5 μM. However, it showed no significant activity against *Bacillus subtilis* or any of the tested Gram-negative bacteria (*Escherichia coli*, *Acinetobacter baumannii*, *Klebsiella pneumoniae*, and *Pseudomonas aeruginosa*), as no MIC values were detected within the tested concentration range (up to 24.8 μM). The control peptide GA-K4AL also demonstrated activity against *S. aureus* (MIC = 2.5 μM) and exhibited a broader antimicrobial spectrum. It effectively inhibited *E. coli* (MIC = 10.1 μM) but was inactive against *B. subtilis* (MIC > 41.8 μM) and the other Gram-negative strains tested.

### 3.5. Inhibition of Cancer Cell Proliferation by Brevinin-1 E8.13 Peptide

**Figure 5:**
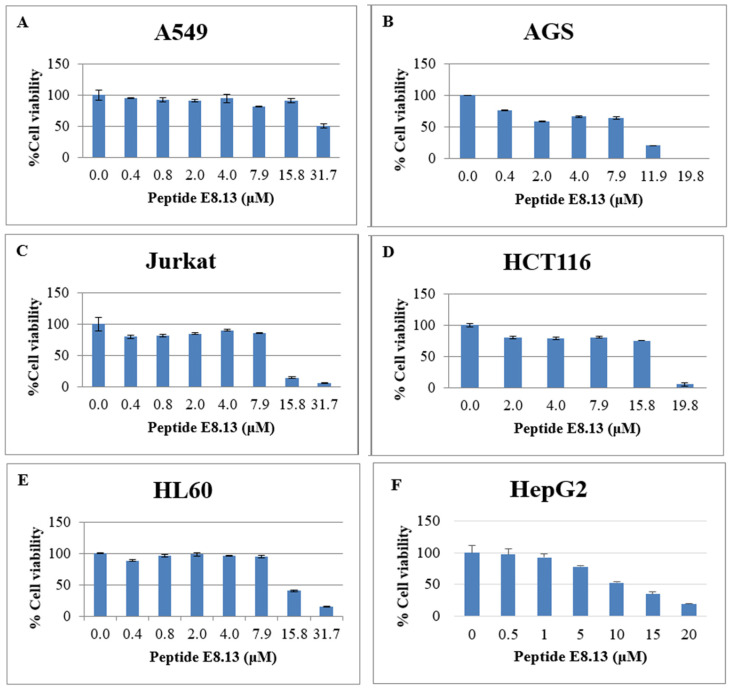
Anti-proliferation activity of brevinin-1 E8.13 peptide on human cancer cell lines. (**A**) A549, (**B**) AGS, (**C**) Jurkat, (**D**) HCT116 and (**E**) HL60, (**F**) HepG2, 0.01% DMSO group (negative control).

The antiproliferative effects of the brevinin-1 E8.13 peptide on various human cancer cell lines were evaluated using the MTT assay at concentrations ranging from 0.4 to 31.7 μM (Figure 5). The peptide was found to inhibit the proliferation of A549 (lung), AGS (stomach), Jurkat (blood), HCT116 (colon), HL60 (blood), and HepG2 (liver) cell lines, with IC_50_ values of 31.6, 7.5, 12.9, 9.2, 14.8, and 11.7 μM, respectively.

**Figure 6:**
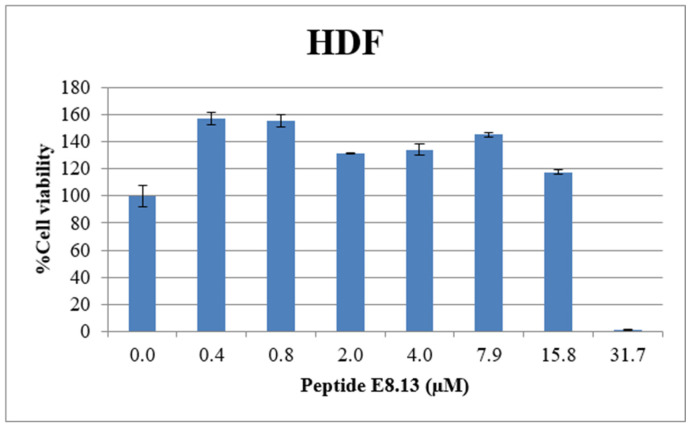
Cytotoxic activity of peptide brevinin-1 8.13 on normal human dermal fibroblast cells (HDF); 0.0 means 0.01% DMSO group (negative control).

The cytotoxic effect of brevinin-1 E8.13 on normal human dermal fibroblast (HDF) cells was also assessed using the MTT assay. Increased cell viability was observed at concentrations between 0.4 and 15.8 μM, while cytotoxic effects became evident at higher concentrations. Complete cell death was recorded at 31.7 μM (Figure 6).

### 3.6. Hemolytic Activity of Brevinin-1 E8.13 Peptide

**Figure 7:**
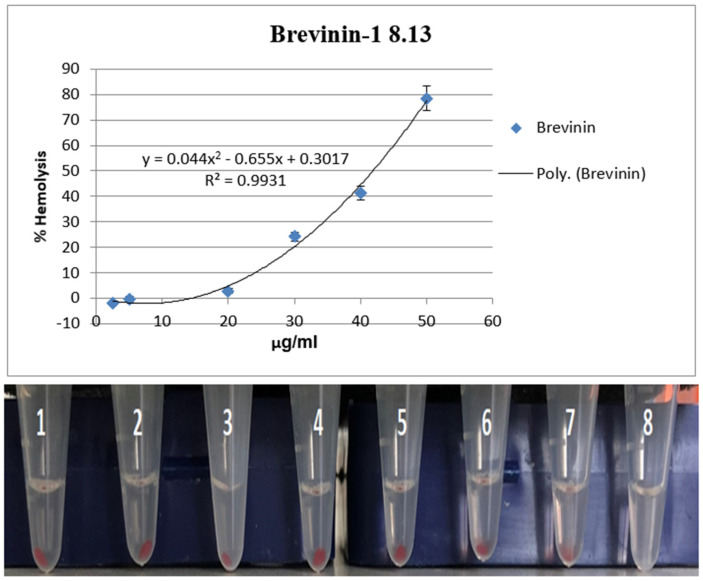
Hemolytic activity of brevinin-1 E8.13 peptide. (1) PBS (negative control). (2) Brevinin-1 E8.13 (0.6 μM). (3) Brevinin-1 E8.13 (1.2 μM); (4) Brevinin-1 E8.13 (4.9 μM). (5) Brevinin-1 E8.13 (7.3 μM). (6) Brevinin-1 E8.13 (9.8 μM). (7) Brevinin-1 E8.13 (12.2 μM). (8) 0.1% Triton X-100 in PBS (positive control).

To assess hemolytic activity, a 2 mL peripheral blood sample was obtained from a healthy volunteer. Red blood cells were isolated by centrifugation, counted, and distributed into 96-well plates at a density of 10^6^ cells/well. The brevinin-1 E8.13 peptide was added at varying concentrations and incubated at room temperature for 1 h. Absorbance was measured at 410 nm to evaluate hemolysis, followed by spectrophotometric analysis across the 400–600 nm wavelength range. The 50% hemolytic concentration (HC_50_) of brevinin-1 E8.13 was determined to be 41.87 μM (Figure 7).

### 3.7. Reduction in Cyp1a1 Expression by Brevinin-1 E8.13 Peptide

**Figure 8:**
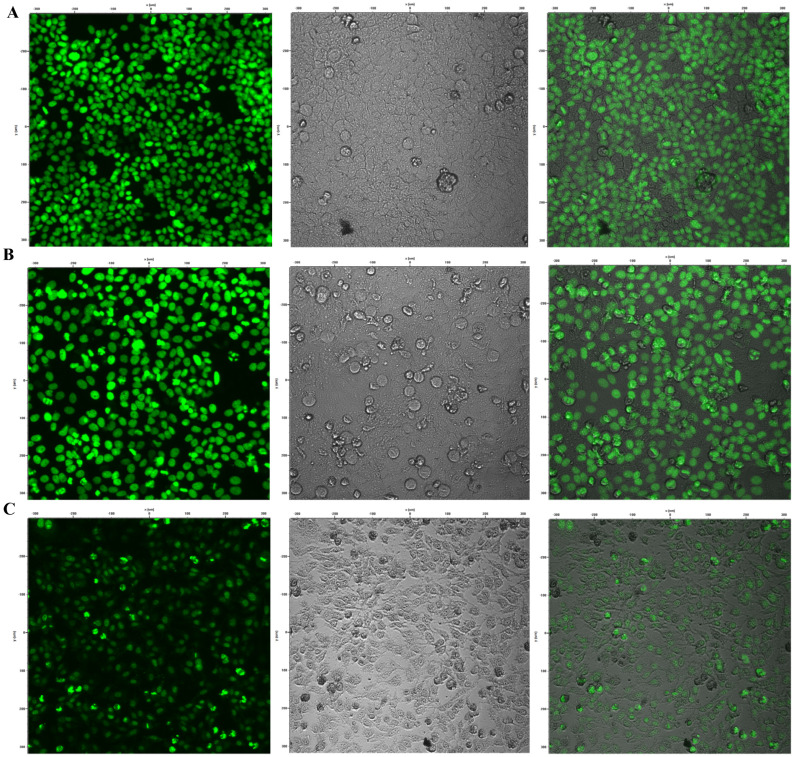
The HepG2 CAFLUX cells after treating with peptide brevinin-1 E8.13; the cells were imaged using a confocal microscope C1si (Nikon, Tokyo, Japan). (**A**) Control sample. (**B**) Cells treated with 5 µM brevinin-1 E8.13. (**C**) Sample cells treated with 10 µM brevinin-1 E8.13 peptide.

**Figure 9:**
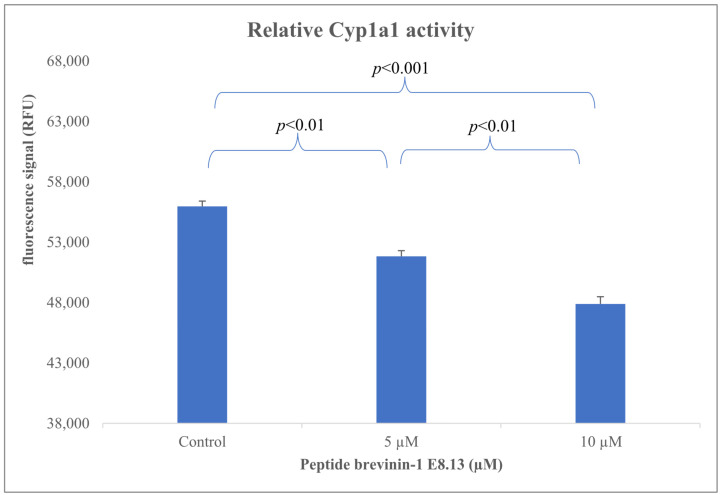
Effect of brevinin-1 E8.13 peptide on Cyp1a1 enzyme activity in HepG2 CAFLUX cells; Chi-squared test.

The expression of the Cyp1a1 gene in HepG2 cells was evaluated using the CAFLUX assay following treatment with brevinin-1 E8.13 peptide. CAFLUX HepG2 cells were incubated with the peptide at concentrations of 5 and 10 μM. A significant reduction in GFP fluorescence intensity was observed, indicating decreased Cyp1a1 expression (Figure 8 and Figure 9). This decrease in gene expression was associated with reduced enzyme activity in HepG2 cells.

## 4. Discussion

Antimicrobial peptides (AMPs) have attracted significant attention due to their potent activity against microbial pathogens and their emerging potential in cancer therapy. The structural and functional diversity of AMPs is largely influenced by the environmental conditions in which the host organisms reside. Amphibians, particularly frog species, typically inhabit moist and microbe-rich environments, which demand robust and adaptable immune defenses. Among these, the secretion of AMPs plays a crucial role, with many peptides undergoing sequence variations at key active sites to enhance their antimicrobial and cytotoxic activities.

Vietnam harbors a rich diversity of Anura species; however, research on AMPs derived from these native amphibians remains limited. This represents both a gap in current knowledge and an opportunity for the discovery of novel bioactive compounds. In recent years, membrane-active peptides have gained prominence as promising anticancer agents due to their rapid cytotoxic effects and low propensity for inducing resistance. These peptides typically act by compromising the integrity of cancer cell membranes, triggering cell death through non-apoptotic and non-classical pathways [10,11,12]. Many naturally derived peptides demonstrate high therapeutic efficacy and selectivity, and their structures can be rationally modified to enhance stability, bioavailability, and potency [13,14]. These findings establish a strong theoretical foundation for the therapeutic development of brevinin-1 E8.13 as a membrane-acting AMP with dual antibacterial and anticancer potential.

In the present study, a novel peptide, brevinin-1 E8.13, was isolated and sequenced from the skin secretion of *Sylvirana guentheri*, a frog species distributed in Ha Giang, Vietnam. Based on the sequence of the mature peptide, it was classified as a member of the brevinin-1 family. Several physicochemical indices of the mature peptide were analyzed, including net charge, percentage of hydrophobic residues, and Boman index. The net positive charge is indicative of cytotoxic potential, while the percentage of hydrophobic residues correlates with the peptide’s ability to bind microbial membranes. It has been established that peptides with a net charge exceeding +8 may also target normal cells [11], and that peptides with a hydrophobic residue content below 30% may be unable to bind effectively to membranes [15,16,17,18]. The brevinin-1 E8.13 peptide was determined to have a net charge of +4 and a hydrophobic residue content of 62%, suggesting suitability as an antimicrobial and anticancer agent. The predicted three-dimensional structure revealed predominantly α-helical regions, which are associated with enhanced stability and membrane-disrupting activity [19].

AMPs typically exert their effects through interactions with multiple targets on the plasma membrane and intracellular structures of bacterial pathogens. Factors influencing lipid polymorphism, such as peptide charge, are critical for membrane interaction. Peptides with a higher positive charge have been associated with greater biological activity [20]. Due to its positive charge, brevinin-1 is capable of altering membrane polymorphism effectively. Additionally, the presence of lysine (LYS) residues, particularly in the central region of the peptide, contributes to interaction with lipid head groups through hydrogen bonding, which facilitates membrane disruption by inducing structural gaps in the lipid bilayer [21].

Based on the brevinin-1 sequence obtained from *Sylvirana guentheri*, the peptide brevinin-1 E8.13 was synthesized using the solid-phase peptide synthesis method. The peptide’s sequence was confirmed by mass spectrometry, and its purity was validated using high-performance liquid chromatography. FTIR spectral analysis revealed several characteristic absorption bands, including the Amide I region at 1638.77 cm^−1^, which is indicative of β-sheet or random coil secondary structures, rather than α-helix. The sharp N–H stretching band near 3277 cm^−1^ suggests strong intermolecular hydrogen bonding, further confirming the presence of peptide backbone interactions. Additional absorption features also support the presence of alkyl chains, aromatic side chains, and intramolecular amide linkages [22].

Brevinin-1 E8.13 contains a high proportion of hydrophobic amino acids such as leucine, valine, phenylalanine, and isoleucine—residues commonly associated with antimicrobial activity. While these residues are often found in α-helical peptides, the secondary structure of brevinin-1 E8.13 is dominated by random coil and β-sheet elements, as shown by FTIR and CD analyses. The abundance of hydrophobic residues contributes to low aqueous solubility but may enhance membrane interaction and antimicrobial potency.

Solubility testing revealed that brevinin-1 E8.13 is only soluble in water at concentrations ≤ 5 mg/mL. This limited solubility may negatively impact its biological efficacy and restrict its applicability in both in vitro and in vivo studies. To address this limitation, future research should explore strategies such as peptide sequence modification, incorporation of hydrophilic residues, or the use of nanocarrier delivery systems to enhance solubility and structural stability.

The synthesized brevinin-1 peptide contained characteristic motifs associated with biological activity, such as C-terminal amidation and a Cys–5aa–Cys motif [23,24]. Compared to a free acid group at the C-terminus, the amidated C-terminus has been shown to enhance antimicrobial activity. The increased positive charge conferred by the amide group strengthens electrostatic interactions between the peptide and negatively charged microbial membranes, improving targeting and binding efficiency. Moreover, C-terminal amidation contributes to membrane interaction mechanisms and enhances secondary structure stability prior to membrane insertion. This modification plays a significant role in both α-helix stability and the lytic activity of peptides [25].

Furthermore, the positive charge conferred by amidation has been linked to increased antimicrobial efficacy. Amidated peptides generally exhibit a higher net positive charge than their non-amidated counterparts, which may account for the increased activity observed in some peptides. According to Mura et al., the interaction between peptides and lipid bilayers is influenced by C-terminal amidation, which facilitates the formation of hydrogen bonds between the carboxyl-terminal region of the peptide and lipid head groups. This interaction affects the behavior of peptides at the water–lipid interface, further supporting the functional significance of amidation in AMP activity [21].

The Cys–5aa–Cys motif, commonly referred to as the “Rana-Box,” is a unique structural feature found in many amphibian-derived antimicrobial peptides (AMPs). This motif includes an intramolecular disulfide bridge located at the C-terminus of the peptide, typically spanning seven to nine amino acid residues. Several functional roles of the “Rana-Box” have been reported and can be summarized as follows: (1) stabilization of the α-helical structure, thereby supporting the biological activity of the peptide; (2) enhancement of structural resistance to enzymatic hydrolysis by proteases such as carboxypeptidase; (3) facilitation of membrane conductance in planar lipid bilayers and induction of potassium ion efflux from bacterial cells; and (4) contribution to the net positive charge of the peptide, which is critical for membrane interaction [26].

Within the brevinin family, particularly brevinin-1, the majority of peptides contain a seven-residue “Rana-Box” that contributes at least two positive charges. These residues are thought to play a critical role in electrostatic interactions between cationic AMPs and the negatively charged membranes of bacterial or cancer cells. The specific membrane-binding model proposed for brevinin-1 peptides suggests that a helical kink enables the N-terminal hydrophobic residues to insert diagonally into the membrane core, while the cationic residues of the “Rana-Box,” positioned on the convex side of the helix, remain exposed at the membrane surface [27].

Brevinin-1 peptides are inherently difficult to express due to their cytotoxicity toward host cells and the high abundance of protease-sensitive residues. However, rational design and structural modifications have been employed to overcome these challenges, thereby improving the feasibility of AMP production for therapeutic applications.

The purified brevinin-1 E8.13 peptide was evaluated for its antibacterial and anticancer properties. Antibacterial activity against Staphylococcus aureus was found to be comparable to that of its GA-K4AL analog. However, brevinin-1 E8.13 demonstrated reduced activity against *Escherichia coli* compared to GA-K4AL, potentially due to differences in peptide length. Membrane binding by peptides has been reported to decrease with reduced peptide length as a result of higher entropic penalties per residue during adsorption [28]. Additionally, the stabilization of transmembrane pore structures depends on peptide length, as adequate alignment with membrane thickness is required. Shorter peptides also exhibit a reduced tendency to form ordered helical secondary structures [29].

Brevinin-1 E8.13 exhibited notable cytotoxicity against various cancer cell lines, which may be attributed to its α-helical regions [30,31]. The Agadir algorithm predicted that 34–36% of the peptide adopts a helical conformation under physiological conditions (37 °C, pH 7.0). The presence of phenylalanine at position 20 (Phe20), a residue rarely observed in brevinin-1 peptides, was also noted [27]. Due to its aromatic side chain, Phe20 may facilitate deeper insertion into lipid bilayers, although it can be electrostatically constrained to the polar headgroup region in anionic membranes under low-ionic-strength conditions [32].

The sharp decline in cell viability observed for brevinin-1 E8.13 within a narrow concentration window (from 7.9 µM to 15.8 µM) aligns with the cooperative threshold-dependent mechanism characteristic of many membrane-active antimicrobial peptides (AMPs). These peptides typically remain inactive until they reach a critical surface concentration on the cell membrane. Beyond this threshold, they rapidly form pores or destabilize the lipid bilayer, causing ion leakage and acute cell lysis in a cooperative manner [33]. Brevinin-1 E8.13 is enriched in hydrophobic and cationic residues, which facilitate strong electrostatic attraction to the negatively charged membranes of cancer cells. Upon exceeding a critical concentration, these peptides can self-assemble into toroidal pores or otherwise destabilize lipid bilayers, resulting in abrupt loss of membrane integrity and rapid cell death. This steep concentration–response profile aligns with models of cooperative membrane disruption observed for other α-helical AMPs [34].

The brevinin-1 E8.13 peptide also induced increased proliferation of human dermal fibroblast (HDF) cells at low concentrations (0.4–15.8 μM). This selectivity is commonly influenced by cancer cells [35], which have been shown to enhance the bioactivity of peptides by stabilizing their secondary structure. Although the hemolytic activity was initially assessed using blood from a single healthy donor, the results suggest a relatively low hemolytic potential, with a calculated HC_50_ value of 41.87 μM. However, future studies should include samples from multiple donors to improve the representativeness and reliability of cytotoxicity assessments. Individual variability in blood type, membrane composition, age, or other biological factors may influence the susceptibility of red blood cells to peptide-induced lysis. Relying on a single donor sample may not capture the full range of biological responses in the human population.

To evaluate the selectivity of brevinin-1 E8.13, we calculated the Selectivity Index (SI) using the formula: SI = IC_50_ (HDF) or HC_50_/IC_50_ (cancer cells) or MIC, where HC_50_ is the peptide concentration causing 50% hemolysis of human red blood cells, MIC is the minimum inhibitory concentration against bacteria, IC_50_ (HDF) is the concentration causing 50% cytotoxicity in normal HDF cells, and IC_50_ (cancer cells) is the corresponding value for cancer cells. An SI < 1 indicates high toxicity to normal cells with poor selectivity, 1 < SI < 10 suggests low to moderate selectivity, and SI > 10 reflects high selectivity. The results indicated that brevinin-1 E8.13 exhibits high selectivity toward *Staphylococcus aureus* over normal cells, highlighting its potential as a safe antimicrobial candidate. The peptide also showed moderate selectivity toward cancer cells, suggesting possible utility in anticancer applications. While the in vitro findings support the anticancer potential of brevinin-1 E8.13 and its selective toxicity toward cancer versus normal cells, further in vivo studies are essential. Animal models will be crucial to assess the peptide’s pharmacokinetics, biodistribution, systemic toxicity, and therapeutic efficacy under physiological conditions. In particular, infection models involving antibiotic-resistant *S. aureus* or xenograft tumor models would be appropriate for evaluating the peptide’s clinical potential.

The cytochrome P450 enzyme Cyp1a1 plays a key role in the metabolic activation of exogenous compounds, including polycyclic aromatic hydrocarbons (PAHs) and heterocyclic amines, into potentially genotoxic metabolites that may contribute to DNA damage and the initiation of liver carcinogenesis [35,36]. Cyp1a1 expression is regulated by the aryl hydrocarbon receptor (AhR), which is activated upon exposure to environmental toxins such as dioxins. Both Cyp1a1 and AhR have been implicated in signaling pathways involved in inflammation and cellular proliferation [37,38].

During its enzymatic activity, Cyp1a1 generates reactive oxygen species (ROS), leading to oxidative stress, DNA damage, and chronic inflammation—factors known to contribute to the development of liver cancer [39]. Elevated expression of Cyp1a1 has been proposed as a potential biomarker for liver cancer progression and metastasis [9,40,41]. Additionally, Cyp1a1 is involved in the metabolism of various chemotherapeutic agents, which can lead to drug resistance or diminished therapeutic efficacy. Modulation of Cyp1a1 expression and activity has thus been suggested as a potential strategy to improve treatment outcomes in liver cancer [42,43]. In this study, the Brevinin-1 E8.13 peptide was found to significantly reduce the expression of the Cyp1a1 gene (Figure 9) (*p* < 0.01). The observed downregulation of Cyp1a1 may be explained by several underlying mechanisms. First, antimicrobial peptides are known to disrupt cell membranes, altering membrane permeability and interfering with intracellular signaling pathways. Second, Brevinin-1 E8.13 may disrupt ionic homeostasis, which may inhibit aryl hydrocarbon receptor activity, which in turn downregulates Cyp1a1 expression. Third, the peptide may induce apoptosis, leading to a cessation of transcription for non-essential genes, including Cyp1a1. To elucidate the precise mechanisms linking Brevinin-1 E8.13 to Cyp1a1 regulation, further comprehensive molecular investigations are warranted.

Cyp1a1 was selected as a representative downstream gene in the aryl hydrocarbon receptor signaling pathway, a well-established pathway known to be activated by environmental toxins and modulated by diverse bioactive compounds, including peptides. The CAFLUX reporter system, which couples green fluorescent protein (GFP) expression to the Cyp1a1 promoter, offers a sensitive and real-time method for detecting AhR-mediated transcriptional activity.

Although Cyp1a1 is not the only gene regulated by AhR, it is widely regarded as a canonical biomarker of AhR activation, driven by its direct transcriptional control Via xenobiotic response elements (XREs). Moreover, multiple studies have shown that bioactive peptides—including antimicrobial peptides—can modulate intracellular signaling cascades such as AhR, NF-κB, and MAPK, thereby influencing gene expression involved in inflammation, detoxification, and apoptosis [44].

## 5. Conclusions

In summary, the present study successfully identified, synthesized, and characterized a novel antimicrobial peptide, brevinin-1 E8.13, from the skin secretion of *Sylvirana guentheri*, a frog species native to Ha Giang, Vietnam. The peptide demonstrated significant antimicrobial activity against *Staphylococcus aureus* and selective cytotoxicity against multiple human cancer cell lines, while showing minimal cytotoxicity toward normal cells at low concentrations. Structural analysis confirmed the presence of conserved AMP motifs, including the C-terminal amidation and the Cys–5aa–Cys “Rana-Box,” both of which are associated with enhanced membrane interaction and stability. Notably, brevinin-1 E8.13 effectively downregulated *Cyp1a1* expression in CAFLUX HepG2 cells, indicating potential utility in modulating pathways relevant to liver carcinogenesis. Taken together, these findings highlight brevinin-1 E8.13 as a promising candidate for further development as a dual-function therapeutic agent with both antimicrobial and anticancer properties.

## Figures and Tables

**Table 1:** The characteristic of interested antimicrobial brevinin-1 E8.13 peptide.

_1_FLGALFKVASKLVPAAICSFSKKC_24_	
Length (residues)	24
Molecular weight	2529.141
Net charge	+4
Hydrophobic residue (%)	62%
Boman Index (kcal/mol)	−0.62 kcal/mol

**Table 2:** The Agadir prediction of brevinin-1 E8.13 peptide.

pH *	Predicted Helical Content	Temperature (°K) **	Predicted Helical Content
5.00	30%	273	69%
6.00	31%	278	71%
6.50	32%	290	54%
7.00	34%	310	36%
7.40	36%	320	30%
7.80	37%	330	25%
8.00	38%	340	21%

* at 310 °K, ** at pH = 7.

**Table 3:** Antibacterial activity of brevinin-1 E8.13 and GA-K4AL.

Treatments	MIC (μM)					
	*S. aureus*	*B. subtilis*	*E. coli*	*A. baumanii*	*K. pneumonia*	*P. aeruginosa*
Brevinin-1 E8.13	1.5	>24.8	>24.8	>24.8	>24.8	>24.8
GA-K4AL	2.5	>41.8	10.1	>24.8	>24.8	>24.8

Note: MIC is defined as the lowest peptide concentration that completely inhibits bacterial growth after 24 h of incubation.

## Data Availability

Data are available upon reasonable request.
